# Color Doppler Imaging Analysis of Ocular Blood Flow Velocities in Normal Tension Glaucoma Patients: A Meta-Analysis

**DOI:** 10.1155/2015/919610

**Published:** 2015-10-29

**Authors:** Shuo Xu, Shouyue Huang, Zhongjing Lin, Wangmin Liu, Yisheng Zhong

**Affiliations:** ^1^Department of Ophthalmology, Ruijin Hospital Affiliated Medical School, Shanghai Jiaotong University, Shanghai 200025, China; ^2^Hubei Provincial Center for Disease Control and Prevention, Wuhan, Hubei 430079, China

## Abstract

*Background*. To evaluate the potential diagnostic value of CDI of retrobulbar hemodynamic changes in NTG patients.* Methods*. Relevant publications which included PSV, EDV, and RI of OA, CRA, NPCA, and TPCA in NTG patients and normal controls measured by CDI were retrieved from the Cochrane Central Register of Controlled Trials, PubMed, the ISI Web of Knowledge, and EMBASE from 1990 to 2014. Subgroup analyses were made based on IOP-lowering medications uses.* Result*. In OA, there was significant decrease of PSV with moderate heterogeneity (*P* < 0.00001, *I* ^2^ = 49%) and significant decrease of EDV with significant heterogeneity (*P* = 0.0005, *I* ^2^ = 87%) in NTG patients. In CRA, similar results of PSV (*P* < 0.00001, *I* ^2^ = 42%) and EDV (*P* < 0.00001, *I* ^2^ = 80%) were detected. Significant decrease of PSV and EDV with significant heterogeneity was also found in both NPCA (*P* < 0.0001, *I* ^2^ = 70%; *P* < 0.0001, *I* ^2^ = 76%; resp.) and TPCA (*P* < 0.00001, *I* ^2^ = 54%; *P* < 0.00001, *I* ^2^ = 65%; resp.). Statistically significant increases of RI were found in CRA (*P* = 0.0002, *I* ^2^ = 89%) and TPCA (*P* = 0.02, *I* ^2^ = 81%) with significant heterogeneities, though RI in OA (*P* = 0.25, *I* ^2^ = 94%) and in NPCA (*P* = 0.15, *I* ^2^ = 86%) showed no statistical changes with significant heterogeneities.* Conclusions*. Ischemic change of retrobulbar hemodynamics is one of the important manifestations of NTG. Hemodynamic parameters measured by CDI might be potential diagnostic tools for NTG.

## 1. Introduction

Normal tension glaucoma (NTG) is a progressive optic neuropathy that mimics primary open angle glaucoma (POAG) but lacks the findings of elevated intraocular pressure (IOP) or other mitigating factors that can lead to optic neuropathy [1]. The main clinical characteristics include consistent normal IOP without treatment, open angle on gonioscope, characteristic glaucomatous optic disc change on ophthalmofundoscope examination, and similar visual field defect with glaucomatous optic nerve abnormality which indicates the damage degree of the optic nerve [2–4]. It is generally accepted that NTG is classified as a subtype of POAG [2]. Originally, NTG was a clinical description from some clinical characteristic of glaucoma. Some of the studies involved in the cornea have indicated that the central corneal thickness (CCT) of NTG patients is thinner than that of normal healthy population and POAG patients, which leads to reduction of IOP reading in NTG patients. Therefore, after adjusting the IOP reading according to the relevant CCT values, IOP may present relatively high in NTG patients. This indicates that the conditions in NTG are similar to those in POAG in spite of difference in CCT [5, 6]. Nevertheless, some evidences have been found recently which suggest the difference between NTG and POAG [3, 7, 8]. For instance, in NTG patients, the shape of defect of VF is different, the decrease of retinal nerve fiber layer is earlier, and the incidence of optic disk hemorrhage is higher significantly than those in POAG patients [9]. All in all, current researches have detected both similarity and difference between NTG and POAG [3, 7, 8]. The similarity and difference between NTG and POAG still coexist concerning pathophysiological mechanisms [1].

If increase of IOP has limited contribution to glaucomatous change, according to the vascular theory of glaucoma, other potential causative factors which are related to vascular abnormality may have influence [10, 11]. Thus, the relationship between retrobulbar hemodynamics and NTG becomes an important subject in the field of glaucomatous researches. It has been hypothesized that vascular spasticity and dysregulation of retrobulbar hemodynamics are connected with NTG, in other words, some cases with normal IOP in POAG [12].

Several techniques such as scanning laser ophthalmoscopy (SLO) [13], Fundus Fluorescein Angiography (FFA) [14], Laser Doppler Flowmetry (LDF) [15–17], Laser Doppler Velocimetry (LDV) [18], Laser Speckle Flowgraphy (LSFG) [19, 20], Pulsatile Ocular Blood Flow (POBF) [21, 22], and Color Doppler Imaging (CDI) [23, 24] have been used to measure retrobulbar hemodynamics in patients with glaucoma. CDI is a technique which evaluates erythrocyte velocity in the large ophthalmic vessels, such as ophthalmic artery (OA), central retinal artery (CRA), and nasal and temporal short posterior ciliary arteries (NPCA and TPCA) [12]. This ultrasonic technique combines synchronous B-ultrasonic wave imaging with color which represents the movement of blood flow based on Doppler frequency shifts. Compared with these techniques above, CDI has its particular advantages. First of all, it is noninvasive. Secondly, it does not require contrast medium. Once more, it is not influenced by poor ocular media. Eventually, this technique has no radiation [23, 24]. CDI is so safe and convenient that it has been used in ophthalmology for more than 20 years.

CDI can provide various velocity parameters of the ocular blood flow (OBF), such as the peak systolic velocity (PSV), the end diastolic velocity (EDV), the mean flow velocity (MFV), systolic mean velocity (Sm), and diastolic mean velocity (Dm). Among them, PSV and EDV are the most extensively used indexes. The resistive index (RI) is also a useful parameter for each retrobulbar vessel, which is calculated as RI = (PSV − EDV)/PSV [12, 25]. A higher value of RI represents greater peripheral vascular resistance, which generally implies deleterious significance [26]. Some studies have made inference that the erythrocyte velocity of OBF measured by CDI may prognosticate the risk of glaucomatous progression, but among various studies the conclusions have not been acquired in the same vessels or with the same measures [15].

Although OBF measurements including CDI have not been currently used on clinic for diagnosis or management in patients with glaucoma yet, several studies have reported significant reduction of PSV or EDV or significant increase of RI in certain vessels of OA, CRA, NPCA, and TPCA in NTG patients by means of CDI measurement [27–30]. Stalmans et al. have made a review and summarized the OBF velocity parameters in different studies by using CDI in healthy populations, OHT patients, and different types of glaucoma patients [31]. However, the studies were conducted in various conditions. Therefore, the results had a lack of comparability among each other and could only be for reference. To the researchers' knowledge, there has been no systematic review published about the evidence of the potential diagnostic value of CDI for NTG. A clear conclusion should be established in this aspect. Therefore, this time we conduct a meta-analysis of the literature for the following purposes: (1) to evaluate the potential diagnostic value of CDI of retrobulbar hemodynamics changes in OA, CRA, NPCA, and TPCA in NTG; (2) to quantify the value of OBF parameter of PSV, EDV, and RI by means of CDI in NTG patients and normal population.

## 2. Materials and Methods

This meta-analysis was conducted according to a predetermined protocol, and the methods used conformed to the Meta-Analysis of Observational Studies in Epidemiology. The related aspects were in agreement with the PRISMA statement [32]. No ethical approval and patient consent were required because all the analyses were based on previous published studies.

### 2.1. Search Strategy

Two researchers seek the research articles from 1990 to 2014 in four electronic databases: the Cochrane Central Register of Controlled Trials, PubMed, the ISI Web of Knowledge, and EMBASE, independently. The search terms were in various combinations from the key words as “color Doppler imaging” or “colour Doppler imaging” or “Doppler ultrasound” or “CDI”, “ocular blood flow” or “retrobulbar blood flow” or “retrobulbar hemodynamics”, and “normal tension glaucoma” or “NTG” or “low tension glaucoma” or “LTG” or “normal pressure glaucoma” or “NPG”. The research articles were limited in the language of English. The researchers also searched the reference lists of included articles for any additional study.

### 2.2. Inclusion Criteria

Inclusive criteria of NTG were similar but slightly different in the included studies. The inclusive criteria were shown, respectively, for each study in Table 4.

In most of the included studies, the following inclusive criteria of normal control subjects were used. Most of normal control subjects were recruited from the persons accompanying the NTG patients; the rest were from the hospital staffs. In most studies, it was clearly declared that the normal control groups were usually age-matched to NTG groups, respectively, in each inclusive study, and those who had a family history of glaucoma, an increased or asymmetrical cup/disc ratio, or any other optic disc structural change were considered as glaucoma suspects and were excluded from normal control subjects. The normal control subjects must have normal IOP (in most of the included studies, IOP was below 21 mmHg) without current medical treatment, including systemic or topical IOP-lowering medications. They had normal, symmetrical optic disc appearance and had no visual field defect. Subjects with a history of neurological or other ophthalmological diseases, ocular traumas, or surgeries were also excluded.

This meta-analysis excluded the studies with the treatment of systemic vasodilator medication.

Published studies were included if they met the following inclusion criteria: (1) studies that were randomized clinical controlled trials or observational studies; (2) studies that compared blood flow velocities with CDI including the parameters of PSV, EDV, or RI in the OA, CRA, NPCA, or TPCA in NTG and normal eyes; (3) studies which were related to the use of CDI measurements performed in supine or sitting position. When multiple studies from the same study population were available, the researchers checked for duplicate analysis to make sure that only the most recent studies were included. More detailed information was provided in the PRISMA checklist (Table 1).

To evaluate eligible inclusion studies, firstly, two researchers screened the titles and abstracts of all search results independently. Secondly, all the citations were classified into one of two groups: (1) relevant; (2) irrelevant. Then, the two researchers retrieved the full articles of relevant citations for further detail to evaluate whether they met the inclusion criteria or not. Only eligible studies were assessed for methodological quality. Finally, two researchers perform a discussion to determine the final selection.

### 2.3. Data Extraction

Before extraction, several procedures were performed so as to convert original data to calculable standard forms. Some studies provided the mean value (*M*) with standard error (SE). The formula $SD=SE\sqrt{n}$ was used to gain standard deviation (SD). In some studies, merging subgroups was necessary. In order to calculate the mean values and SDs of the parameters in combined groups, the formulas

(1) M=N1M1+N2M2N1+N2,SD=N1−1SD12+N2−1SD22+N1N2M1+M2−2M1M2/N1+N2N1+N2−1

were, respectively, used by the researchers.

The following detailed information was extracted from the published studies into a customized reporting form: (1) study information (study name, first author's name, year of publication, and publication journal); (2) basic study data (country of origin, number of enrolled subjects, mean with SD of age of subjects, gender ratio, and ethnic composition); (3) quality-related data (inclusive criteria of NTG, type of CDI equipment, type of treatment, and subjects' measurement position); (4) outcome measures data (mean value with SD of CDI measurement parameters). Two researchers carried out the data extraction independently. Disagreements were resolved by discussion with one another.

### 2.4. Outcome Measures

All studies compared blood flow velocities including the parameters of PSV and EDV measured by CDI in the OA, CRA, NPCA, and TPCA as major outcomes. PSV (cm/s) was defined as the highest blood flow velocity achieved during systole and was calculated from the frequency of the peak in the Doppler-shifted waveform. EDV (cm/s) was defined as the lowest velocity occurring during diastole and was calculated from the frequency of the trough in the Doppler-shifted waveform [33]. RI was calculated for each vessel by the formula RI = (PSV − EDV)/PSV which has been widely used and established by Planiol et al. [25].

### 2.5. Statistical Analysis

This meta-analysis was performed by using RevMan software (Review Manager, Version 5.3; The Cochrane Collaboration, 2014) and STATA 13.0. Mean difference (MD) was calculated, respectively, for the continuous outcomes (parameter of PSV, EDV, and RI) with 95% confidence intervals (95% CIs) in each single parameter analysis.

Results of each analysis were shown in forest plots. Chi-square based Cochran's statistics and inconsistency statistic (*I* ^2^) were used to determine heterogeneity (the variation in findings not compatible with chance alone) across studies, which indicates the proportion of the variability across studies due to heterogeneity instead of sample error and quantifies heterogeneity irrespective of the number of studies [34, 35]. In this meta-analysis, statistically significant heterogeneity was defined when *P*_heterogeneity_ < 0.10 and *I* ^2^ > 50%. Moderate heterogeneity was defined when *I* ^2^ ≤ 50%. When substantial heterogeneity (*I* ^2^ > 50%) was found in single parameter analysis, the random effects model was adopted (in this meta-analysis, substantial heterogeneities of the “mixed or not available” subgroups were ignored because studies in these subgroups have already had clinical heterogeneities. Once substantial heterogeneity which meant *I* ^2^ > 50% was found in any group among total group, treated subgroup, or treated subgroup except the “mixed or not available” subgroup, the random effects model would be used in single parameter analysis) [36]. Otherwise, the fixed effects model was used [37]. Funnel plots for each single parameter analysis were provided to estimate publication bias. All these statistics and figures above were provided by RevMan v5.3, automatically.

### 2.6. Risk of Bias Assessment

In order to detect publication bias, funnel plot was provided and Begg rank correlation test and Egger linear regression test were performed by Stata 13.0 for each analysis [38, 39]. To detect and classify the remaining bias, a self-made risk of bias scale was used. Fail-safe numbers (*N*_fs_) were also calculated, respectively, to determine the stability of the results when the results have statistically significant difference. Since RevMan v5.3 does not have the function of calculating *N*_fs_ yet, the researchers calculated *N*_fs_ manually with the help of Microsoft Excel. The researchers referred to the formulas ①  *N*_fs_ = (∑*t*/1.645)^2^ − *k*  ②  $t=\left({\overset{-}{x}}_{1}-{\overset{-}{x}}_{2}\right)/\sqrt{S_{c}^{2}(1/n_{1}+1/n_{2})}$  ③  *S*_*c*_^2^ = [(*n*_1_ − 1)*S*_1_^2^ + (*n*_2_ − 1)*S*_2_^2^]/(*n*_1_ + *n*_2_ − 2) according to the method of calculating *N*_fs_ of continuous outcome in meta-analysis [40].

Since previous studies showed that topical antiglaucoma medications may have effect on retrobulbar hemodynamics by means of increasing ocular blood flow velocity and decreasing RI [33, 41, 42], this may contribute to the heterogeneity across studies. Subgroup analyses were performed according to whether NTG patients used IOP-lowering medications and were divided as treated subgroup, untreated subgroup, and the rest (treated and untreated patients mixed or information not available in the studies). The treated subgroup included NTG patients with current IOP-lowering medications treatment. The untreated subgroup included NTG patients having previous IOP-lowering medications treatment with a wash-out period of at least 3 weeks, NTG patients who never receive IOP-lowering medications treatment, or patients with a first-time diagnosis of NTG.

## 3. Results

### 3.1. Study Characteristics

**Figure 1:**
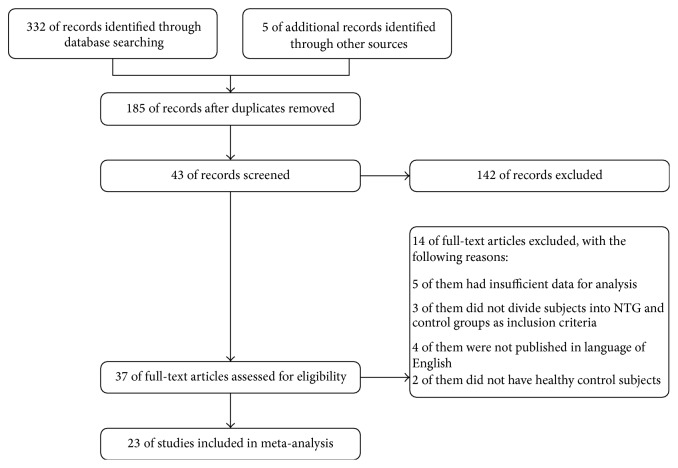
Flow diagram of the study selection process.

Figure 1 (made by RevMan v5.3) shows the flowchart of the selection process to determine eligible studies. The researchers reviewed the full text of 37 studies from 332 studies searched from databases and other sources, and finally 23 studies were included in this meta-analysis.

Sixteen studies (69.57%) were conducted in Europe (4 in Germany, 4 in Portugal, 3 in Italy, 2 in UK, 2 in Belgium, and 1 in Switzerland); 3 of them (13.04%) were in America (2 in Canada and 1 in USA) and the remaining 4 of them (17.39%) were in Asia (2 in China, 1 in Japan, and 1 in Turkey). Twenty-one of the included studies (91.30%) provided detailed age information of studied populations. The age ranges of the population of NTG patients recruited were 18 years and over. In the majority of the included studies, the mean age of NTG patients was over 50 years (*n* = 19, 82.61%). The gender ratios of the studied population in the included studies were various from each other and the data were not available in 11 of the studies (47.83%).

In 15 of the studies (65.22%), CDI examinations were undertaken in the supine position (among them, 2 of the studies performed CDI in both supine and upright positions. One study showed no significant difference between postural changes in retrobulbar hemodynamics, while 1 study detected significant difference. So we just chose the results in the supine position of these 2 studies.). In 1 study (4.35%), CDI examinations were performed in the sitting position of reclining at 60 degrees. The remaining 7 studies (30.43%) did not provide clear postural information. In 7 of the included studies (30.43%), NTG patients were untreated, including patients having previous IOP-lowering medications treatment with a wash-out period of at least 3 weeks, patients never receiving IOP-lowering medications treatment, or patients with a first-time diagnosis of NTG. Eight of the included studies (34.78%) indicated clearly that NTG patients in these studies continued current IOP-lowering medications treatment. In the remaining 8 studies (34.78%), treated and untreated patients were mixed or authors did not mention treatment information clearly. Clinical heterogeneity might be caused in various aspects, including the individual characteristics of the patients, different age or gender composition, inclusive criteria of NTG, CDI equipment, postural position change, or different treatment protocols. Since these factors were not reported consistently in the studies, they were not analyzed in this meta-analysis. More detailed characteristics of the 23 included studies were calculated and provided in Tables 2 [Table tab3] [Table tab4]–5.

### 3.2. Risk of Bias

There was significant asymmetry in the funnel plot of RI in OA. Funnel plots of the remaining analyses were almost symmetrical. Begg rank correlation test showed that conclusion of RI in OA might be influenced by potential publication bias (*P* = 0.005), while no publication bias was detected by Egger linear regression test in each conclusion of analysis. That meant the result of RI in OA was not true and not reliable (but we also reported the result below). Funnel plots and the results of Begg rank correlation test and Egger linear regression test for each part of the analyses were not provided because of the space and picture limitations.

**Figure 2:**
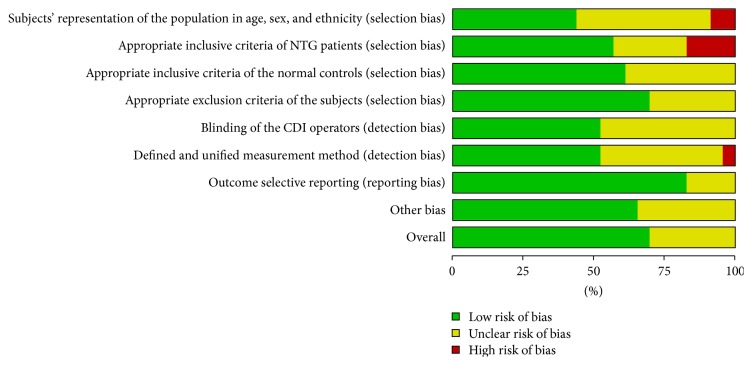
Risk of bias rate of each item.

**Figure 3:**
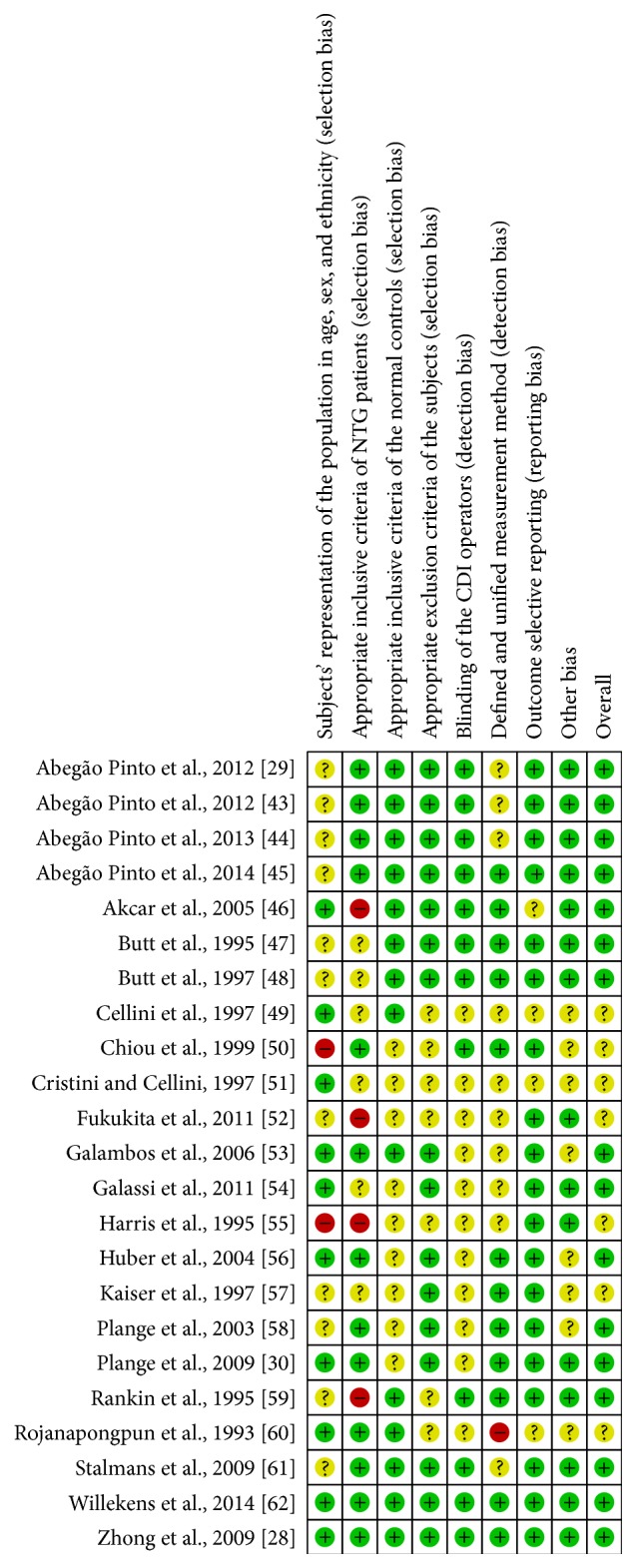
Risk of bias summary of each item.

The remaining bias within studies was classified by a risk of bias scale made by ourselves. In 23 included studies, 15 studies (69.57%) were evaluated as having a low risk of bias and 8 (30.43%) were evaluated as having a moderate risk of bias overall. High risk of bias was most common for selection bias (subjects' representation of the population, 8.70%, and inclusive criteria of NTG patients, 17.39%). Moderate risk of bias ranged from 17.39% to 47.83% and was relatively equally located in each item, especially in items of subjects representation of the population (47.83%), inclusive criteria of the normal controls (39.13%), blinding of the operators (47.83%), and measurement method (43.48%). Detailed information of each item was provided in Figures 2 and 3.

### 3.3. Meta-Analysis Results

**Figure 4:**
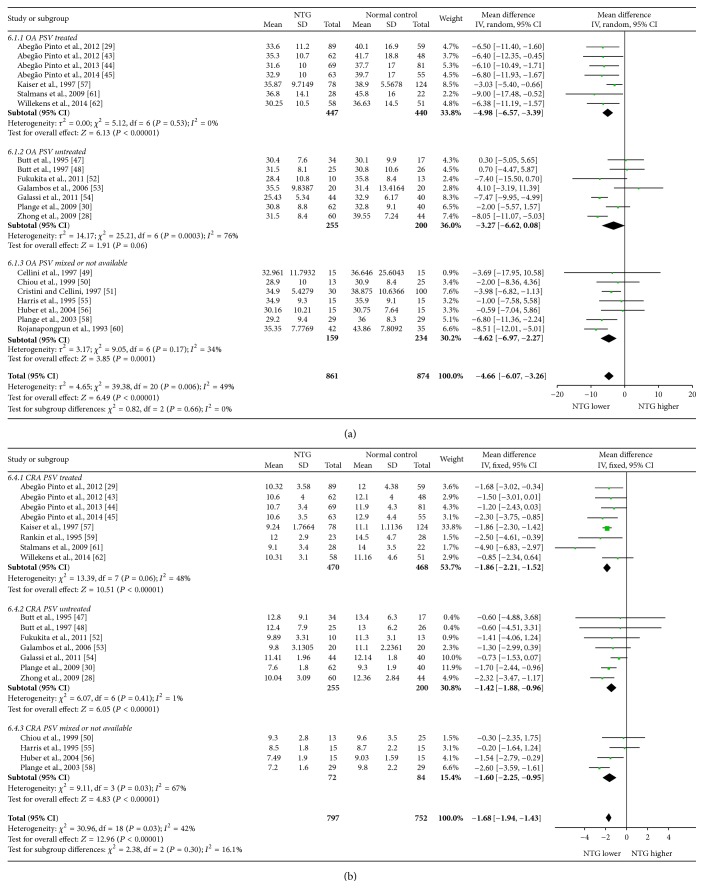
Forest plot of PSV in OA and CRA.

**Figure 5:**
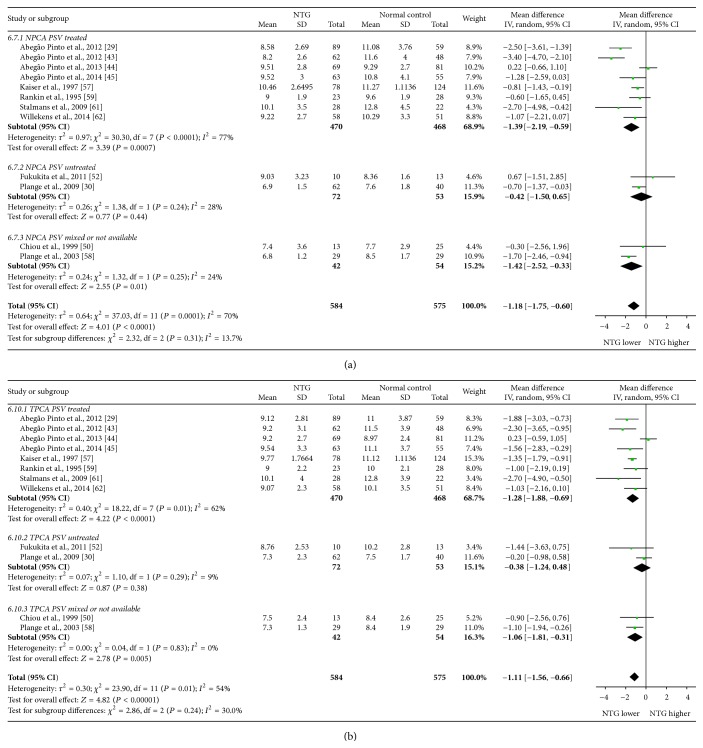
Forest plot of PSV in NPCA and TPCA.

**Figure 6:**
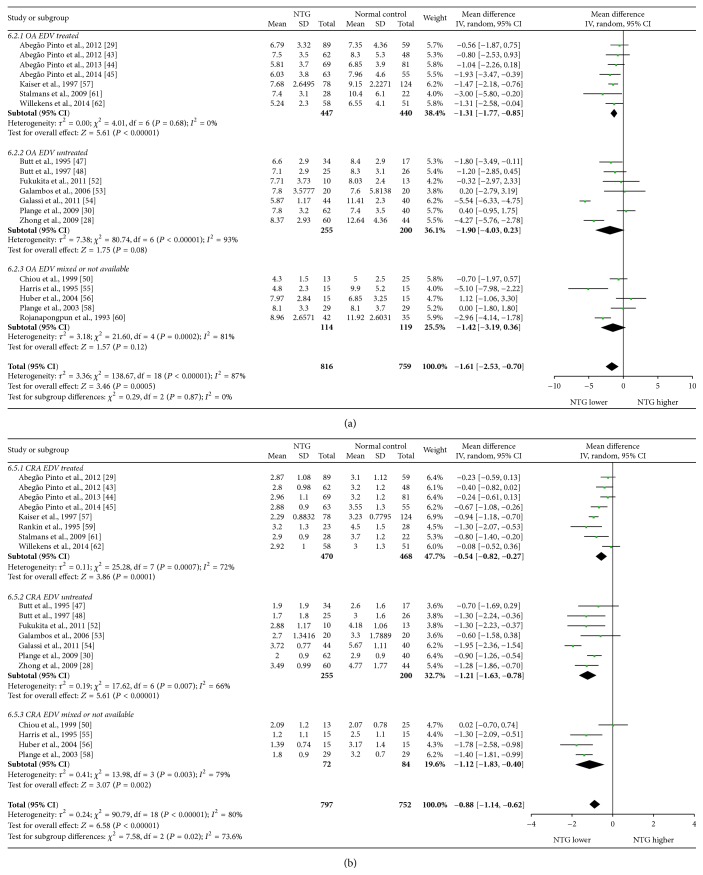
Forest plot of EDV in OA and CRA.

**Figure 7:**
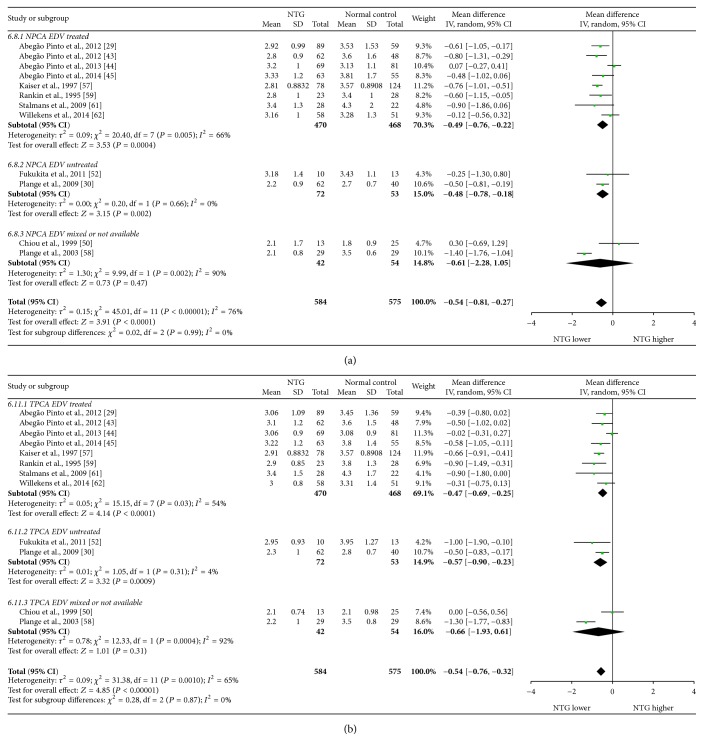
Forest plot of EDV in NPCA and TPCA.

**Figure 8:**
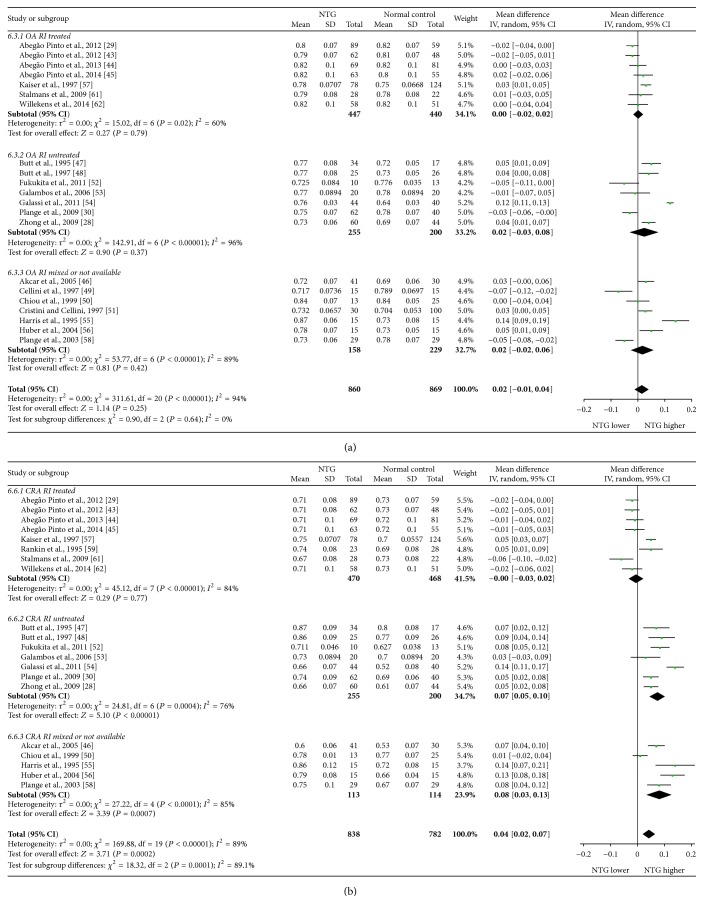
Forest plot of RI in OA and CRA.

**Figure 9:**
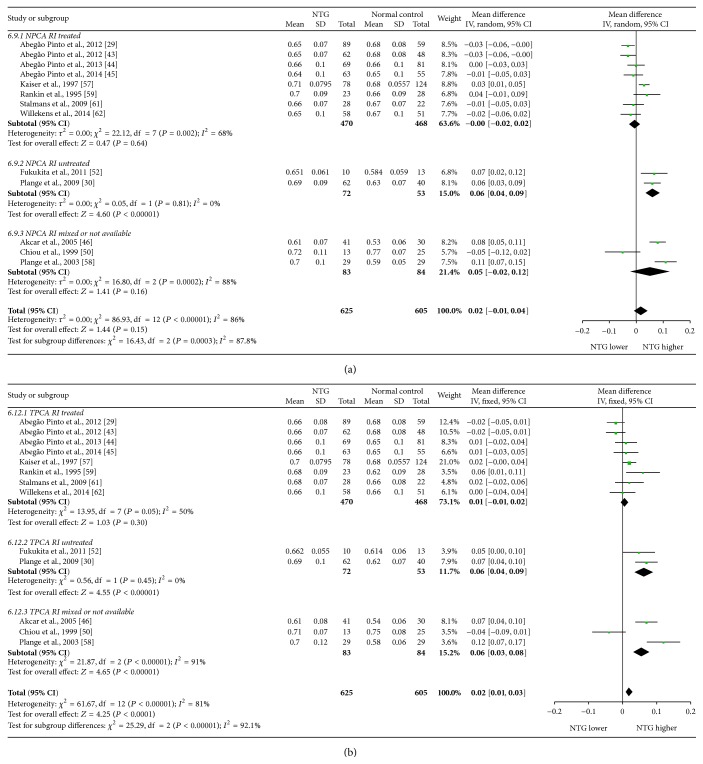
Forest plot of RI in NPCA and TPCA.

Mean differences (MDs) with 95% confidence intervals (95% CIs) of PSV, EDV, and RI of OA, CRA, NPCA, and TPCA were shown in forest plots (Figures 4–9). The green dots represented MDs and the whiskers which extend from the dots represented the 95% CIs of MDs. Values on the left side of the vertical line marked 0 indicated that parameters in NTG patients were smaller than normal controls while values on the right side of the vertical line indicated the increase of parameters in NTG patients. Whiskers of 95% CIs which did not cross the vertical line indicated that the results had statistically significant difference at the level of *α* = 0.05.

Statistically significant decreases were found in PSV in OA, CRA, NPCA, and TPCA, respectively, with the MDs of −4.66 cm/s (95% CI: −6.07 to −3.26, *P* < 0.00001, and *N*_fs_ = 624.1325), −1.68 cm/s (95% CI: −1.94 to −1.43, *P* < 0.00001, and *N*_fs_ = 851.1949), −1.18 cm/s (95% CI: −1.75 to −0.60, *P* < 0.0001, and *N*_fs_ = 239.6319), and −1.11 cm/s (95% CI: −1.56 to −0.66, *P* < 0.00001, and *N*_fs_ = 250.4296). There were moderate heterogeneities in PSV in OA (*P*_heterogeneity_ = 0.006, *I* ^2^ = 49%) and CRA (*P*_heterogeneity_ = 0.03, *I* ^2^ = 42%) while there were significant heterogeneities in NPCA (*P*_heterogeneity_ = 0.0001, *I* ^2^ = 70%) and TPCA (*P*_heterogeneity_ = 0.01, *I* ^2^ = 54%).

Subgroup analysis of PSV demonstrated that there were significant heterogeneities in treated subgroups in NPCA (*P*_heterogeneity_ < 0.0001, *I* ^2^ = 77%) and TPCA (*P*_heterogeneity_ = 0.01, *I* ^2^ = 62%), while moderate heterogeneities were found in OA (*P*_heterogeneity_ = 0.53, *I* ^2^ = 0%) and CRA (*P*_heterogeneity_ = 0.06, *I* ^2^ = 48%). In untreated subgroups, significant heterogeneity was only found in OA (*P*_heterogeneity_ = 0.0003, *I* ^2^ = 76%), while moderate heterogeneities were found in CRA (*P*_heterogeneity_ = 0.41, *I* ^2^ = 1%), NPCA (*P*_heterogeneity_ = 0.24, *I* ^2^ = 28%), and TPCA (*P*_heterogeneity_ = 0.29, *I* ^2^ = 9%). Heterogeneities between subgroups were unapparent in each of these 4 vessels (OA: *P*_subgroup_ = 0.66, *I* ^2^ = 0%; CRA: *P*_subgroup_ = 0.30, *I* ^2^ = 16.1%; NPCA: *P*_subgroup_ = 0.31, *I* ^2^ = 13.7%; TPCA: *P*_subgroup_ = 0.24, *I* ^2^ = 30.0%).

The forest plots of PSV in each vessel were provided in Figures 4 and 5.

NTG patients had statistical reduction of EDV in OA, CRA, NPCA, and TPCA, with the MDs of −1.61 cm/s (95% CI: −2.53 to −0.70, *P* = 0.0005, and *N*_fs_ = 763.2090), −0.88 cm/s (95% CI: −1.14 to −0.62, *P* < 0.00001, and *N*_fs_ = 1456.5108), −0.54 cm/s (95% CI: −0.81 to −0.27, *P* < 0.0001, and *N*_fs_ = 287.1655), and −0.54 cm/s (95% CI: −0.76 to −0.32, *P* < 0.00001, and *N*_fs_ = 281.1861), respectively. Statistical significant heterogeneities were found in each vessel (OA: *P*_heterogeneity_ < 0.00001, *I* ^2^ = 87%; CRA: *P*_heterogeneity_ < 0.00001, *I* ^2^ = 80%; NPCA: *P*_heterogeneity_ < 0.00001, *I* ^2^ = 76%; TPCA: *P*_heterogeneity_ = 0.001, *I* ^2^ = 65%).

Subgroup analysis of EDV showed that, in treated subgroups, significant heterogeneities were found in CRA (*P*_heterogeneity_ = 0.0007, *I* ^2^ = 72%), NPCA (*P*_heterogeneity_ = 0.005, *I* ^2^ = 66%), and TPCA (*P*_heterogeneity_ = 0.03, *I* ^2^ = 54%), while moderate heterogeneity was found in only OA (*P*_heterogeneity_ = 0.68, *I* ^2^ = 0%). In untreated subgroups, significant heterogeneities were found in OA (*P*_heterogeneity_ < 0.00001, *I* ^2^ = 93%) and CRA (*P*_heterogeneity_ = 0.007, *I* ^2^ = 66%), while moderate heterogeneities were found in NPCA (*P*_heterogeneity_ = 0.66, *I* ^2^ = 0%) and TPCA (*P*_heterogeneity_ = 0.31, *I* ^2^ = 4%). Significant heterogeneities between subgroups were detected in CRA (*P*_subgroup_ = 0.02, *I* ^2^ = 73.6%). Heterogeneities between subgroups were unapparent in each of the OA (*P*_subgroup_ = 0.87, *I* ^2^ = 0%), NPCA (*P*_subgroup_ = 0.99, *I* ^2^ = 0%), and TPCA (*P*_subgroup_ = 0.87, *I* ^2^ = 0%).

The forest plots of EDV in each vessel were provided in Figures 6 and 7.

As for RI, there were statistical increases in CRA (MD: 0.04, 95% CI: 0.02 to 0.07, *P* = 0.0002, and *N*_fs_ = 741.4045) and TPCA (MD: 0.03, 95% CI: 0.00 to 0.05, *P* = 0.02, and *N*_fs_ = 91.6852). No significant changes were found in OA (MD: 0.02, 95% CI: −0.01 to 0.04, *P* = 0.25) and NPCA (MD: 0.02, 95% CI: −0.01 to 0.04, *P* = 0.15). High heterogeneities were found in all these vessels (OA: *P*_heterogeneity_ < 0.00001, *I* ^2^ = 94%; CRA: *P*_heterogeneity_ < 0.00001, *I* ^2^ = 89%; NPCA: *P*_heterogeneity_ < 0.00001, *I* ^2^ = 86%; TPCA: *P*_heterogeneity_ < 0.00001, *I* ^2^ = 81%).

Subgroup analysis of RI showed that, in treated subgroups, heterogeneities were significant in OA (*P*_heterogeneity_ = 0.02, *I* ^2^ = 60%), CRA (*P*_heterogeneity_ < 0.00001, *I* ^2^ = 84%), and NPCA (*P*_heterogeneity_ = 0.002, *I* ^2^ = 68%), while heterogeneity was moderate in only TPCA (*P*_heterogeneity_ = 0.05, *I* ^2^ = 50%). In untreated subgroups, heterogeneities were significant in OA (*P*_heterogeneity_ < 0.00001, *I* ^2^ = 96%) and CRA (*P*_heterogeneity_ = 0.0004, *I* ^2^ = 76%), while they could be ignored in NPCA (*P*_heterogeneity_ = 0.81, *I* ^2^ = 0%) and TPCA (*P*_heterogeneity_ = 0.45, *I* ^2^ = 0%). There were high heterogeneities between subgroups in CRA (*P*_subgroup_ = 0.0001, *I* ^2^ = 89.1%), NPCA (*P*_subgroup_ = 0.0003, *I* ^2^ = 87.8%), and TPCA (*P*_subgroup_ = 0.002, *I* ^2^ = 84.3%), while there was moderate heterogeneity between subgroups in OA (*P*_subgroup_ = 0.64, *I* ^2^ = 0%).

The forest plots of RI in each vessel were provided in Figures 8 and 9.

In addition, there were, respectively, only 2 studies included in untreated subgroup in NPCA and TPCA of each single parameter analysis, which made the results of untreated subgroup in NPCA and TPCA unstable and unreliable.

## 4. Discussion

NTG is an optic neuropathy within the glaucoma family which shares many of the same pathologic characteristics as POAG with cupping of the optic disc and visual field loss resembling that seen in other types of chronic open angle glaucoma except for the fact that a statistically elevated IOP cannot be detected [63]. Once, it was thought uncommon. But several recent population studies both in the United States and abroad have indicated that NTG accounts for between 20% and 40% of all open angle glaucoma [64–68]. The role of IOP has long been questioned in the development of NTG. Several studies about asymmetric NTG showed greater glaucomatous damage in the eye with the higher IOP, which seemed to support the theory that NTG was pressure sensitive [69, 70]. However, others found that only 27.66% of NTG patients with asymmetric visual field defects had a mean IOP difference of more than 1 mmHg between two eyes [71]. This suggested that IOP had limited impact of NTG development. If there is no significant elevation of IOP, vascular abnormality might result in glaucomatous damage according to the vascular theory of glaucoma [10, 11].

This meta-analysis showed that in NTG patient PSV and EDV decreased in each of the OA, CRA, NPCA, and TPCA, while RI statistically significantly increased in CRA and TPCA. It is generally considered that the OA is the main source of blood supply to the optic nerve; the SPCAs (NPCA and TPCA) are the main source for optic nerve head (ONH) perfusion, with small contributions from the pial vessels and CRA, and the blood supply of retina mainly from the CRA [72]. And all CRA, NPCA, and TPCA are branches of the OA. Therefore, the hemodynamic parameters of the OA, CRA, NPCA, and TPCA can show the blood supply conditions of the ONH and retina. The parameter of PSV refers to the highest blood flow velocity achieved during systole and is calculated from the frequency of the peak in the Doppler-shifted waveform. It reflects the strength of vessel perfusion. EDV refers to the lowest velocity occurring during diastole and is calculated from the frequency of the trough in the waveform. It reflects the blood perfusion of distal organs [33]. Increase of vascular resistance has more influence on diastolic blood flow velocity than systolic blood flow velocity [73]. Therefore, EDV is a more sensitive indicator of increased downstream impedance than PSV. A simultaneous increase in both EDV and PSV may reflect an increase of total volumetric flow, and the opposite conclusions may apply to a simultaneous decrease in EDV and PSV [74]. Alternatively, an increase in PSV may be caused by a local vasoconstriction within a vessel, which may result in increased blood flow velocities without any change in volumetric flow [75]. RI is reported to be highly correlated to downstream vascular resistance in vessels [76]. But it is not equivalent to vascular resistance because it depends on both vascular resistance and vascular compliance. RI can highly represent vascular resistance only when the vascular compliance is high. Nevertheless, higher RI with lower EDV can be considered as an increase of vascular resistance [77, 78].

According to the above explanations, this meta-analysis indicated that, in NTG patients, retrobulbar blood flow has a reduction of both systolic proximal vessel perfusion and diastolic distal organs perfusion, and downstream vascular resistance significantly increases in CRA and TPCA. Hypothesis has existed for more than half a century that glaucoma is the expression of stasis in the venous system and eye capillaries and has its origin in organic vascular changes with spastic factor or in neurovascular function changes [79]. Ischemia caused by various vascular factors may play a major role in the glaucomatous damage. Several recent studies showed that blood pressure and ocular perfusion pressure were risk factors with high correlation for the incidence and progression of glaucoma. The Barbados Eye Study [80] reported that low systolic blood pressure doubled the risk for glaucoma incidence and those with the lowest 20% of diastolic perfusion pressure had a 3.3 times risk of developing glaucoma. The Proyecto VER Study [81] reported that patients with a diastolic perfusion pressure as low as 45 mmHg were 3 times more likely to develop glaucoma compared with those who had a diastolic perfusion pressure of 65 mmHg. A recent study presented a possible theory, which was more related to vascular factors, describing pathways depending on low ocular perfusion pressure (OPP) [82]: low OPP (which could be caused by vascular factors and IOP increase) might decrease mitochondria energy state in the retinal ganglion cells (RGCs) or reduce nutrient flow of the RGC axons. Then, oxidative stress due to reactive oxygen species (ROS) led to RGC apoptosis. Low OPP may also cause abnormal vascular autoregulation and neurovascular coupling, which also functioned on RGC losses. The result of this meta-analysis is a strong support of the vascular theory of glaucoma and it suggests that retrobulbar ischemia has influence on not only the ONH but also the retina. Based on vascular theory of glaucoma [82], we have the reason to suppose that reduction of blood flow caused by vascular abnormality in the CRA probably leads to ischemia of the inner retina on NTG process and finally results in apoptosis of ganglion cells. Low RNFL thickness in NTG patients is exactly the manifestation of apoptosis of ganglion cells.

There are several limitations of this meta-analysis to be considered. Firstly, the included studies are essentially observational studies which means there are no intervention measures from researchers in the studies. Compared with experimental studies, observational study is more likely to be affected by bias risks. Secondly, only studies published in English were searched and selected, which might lead to a risk of English language bias. Furthermore, in a part of the patients there existed various preexisting comorbidities whose detailed information was not completely given in the studies, which might possibly affect blood flow. Finally, different characteristics of the study populations, different inclusion criteria of NTG patients and normal controls, different study eye selection strategy, different medication treatment conditions, and different CDI measurement equipment, operators, and process might also have influence on the conclusion. They were inevitable because it was too various to make subgroup analysis.

Publication bias may have distorted our conclusion. Only one significant publication bias was found in a single analysis (RI in OA). Funnel plots were almost symmetrical except for the analysis of RI in OA. The final results underwent Beggar's and Egger's tests and there showed only one publication bias in the analysis of RI in OA. However, publication bias remains in other analyses because studies that report nonsignificant results have less possibility to be published than those that report statistically significant results. Furthermore, we did not include unpublished studies from conference abstracts, dissertations, or pharmaceutical companies.

There were considerable heterogeneities between the included studies. Heterogeneities may be caused by medication treatment conditions, patient characteristics, sample size, diagnostic criteria, and operator experience. In addition, different CDI devices may also lead to heterogeneity in this meta-analysis because of variable sensitivities, specificities, and positive predictive values. We conducted subgroups analyses according to whether the patients had medication treatment and reduced a part of the heterogeneities but there were still great heterogeneities. The remaining factors were too various to make subgroup analysis and their data types were not appropriate to perform meta-regression which explained some of the heterogeneities.

This meta-analysis was performed to assess the OBF changes in NTG populations by means of CDI. Contemporarily, CDI of retrobulbar vessels is limited to the measurement of various blood flow velocity parameters in general. Blood flow through a vessel can be calculated by *Q* = MFV · *π* · *R* ^2^ (MFV is the abbreviation of mean flow velocity, which can be measured by CDI; *R* represent the diameter of the vessel). But, since CDI method has a lack of reliable quantification of accurate diameter in orbital vessels in vivo [1, 33], it cannot reflect actual OBF directly. Hence, CDI results cannot be interpreted as blood flow values, unless the diameter of the vessels is also measured. There are now advances in CDI analysis software that allows three-dimensional reconstructions of blood vessels and flow. Estimation of the vessel diameter from these reconstructions allows calculation of volumetric flow based on volumetric measurements. Several studies have attempted to measure absolute blood flow in certain vessels, but limited data on reproducibility may currently limit the use of this technique in clinical studies [83–86].

CDI technique also has several other limitations which may make the results unreliable. First of all, because CDI measurement highly depends on probe position and Doppler angle (*θ*), the velocity of the blood flow can be described in the formula *u* = Δ*f* · *C*/(2*f*_0_ · cos⁡*θ*), in which *u* is the velocity of the blood flow, *f*_0_ means the frequency of the sound source, and *C* means the sound velocity in the medium. When the probe is almost parallel to a vessel (cos⁡*θ* = 1), there is the least possible influence of Doppler angle uncertainty. However, because of the reflection effect of the sound waves by vessel wall, using very small angles may face technical difficulties [31]. In fact, in consideration of various factors, Doppler angles between 30° and 60° are the most frequently used angles in clinical practice. Errors in estimating the Doppler angle always cause the result changes. In particular, when the Doppler Angle exceeds 60°, small errors may lead to large changes in the velocity of the blood flow [87]. All results of direct measurement, such as PSV, EDV, and MFV, can be influenced by Doppler angle, whereas RI is independent of the Doppler angle because changes in Doppler angle have a concomitant effect on PSV and EDV when we calculate RI. In a word, errors in estimating the Doppler angle may reduce the repeatability of results. Secondly, there is possibility to confuse short ciliary artery with long ciliary artery and one ciliary artery with several ciliary arteries because of the small size of these vessels, the proximity between them, and the variability in number and position [72]. Once again, the reproducibility of CDI among different operators is acceptable in certain vessels or parameters but not quite good in others. A study showed that coefficient of variations (COVs) for interoperators for PSV, EDV, and RI in OA reached up to 8.2%, 25.5%, and 6.2%, respectively, and 19.3%, 25%, and 6.3% in CRA, respectively [88]. Nevertheless, COVs for the same operator for PSV, EDV, MFV, and RI in OA were 7.4%, 8.5%, 8.2%, and 1.3%, respectively [72]. Moreover, individual variations on vascular organizations and tissue densities might have great influence on measurement results. Finally, when IOP is higher than 45 mmHg, the velocity of OBF hardly changes because much of blood flow in OA cannot perfuse the eye. In this situation, it is difficult to explain the measurement results of velocity parameters measured by CDI [89]. In spite of these defects, velocity parameters of OBF measured by CDI might still be highly relative indexes to reflect OBF in vivo [73, 90].

A variety of other techniques which have been used to determine OBF, such as FFA, LDV, LDF, LSFG, and POBF, encounter a similar problem and have their own limitations when they are used in vivo. Furthermore, the OBF measurements on humans in general have quite a lot of noise and the results are highly variable. These make it difficult to determine the fundamental characteristics of OBF and make the measurement results variable and uncertain. In fact, to date, no wide consensus has been reached as to which single technique should be used to asses OBF comprehensively and how the results should be explained. Different measurement techniques provide different details of vascular parameters and can be explained differently. None of the methods has been sufficiently standardized or validated externally for humans as gold standard in clinical practice yet [1, 12]. Among the available existing techniques to assess the vascular component in glaucoma, CDI seems to be the most advisable because of its noninvasive and acceptable reproducibility compared to other techniques [72].

In a word, this meta-analysis showed that PSV and EDV decreased in OA, CRA, NPCA, and TPCA, and RI increased in CRA and TPCA in NTG patient.

It seems that the OBF changes in NTG development are common. CDI could be a potential technology for diagnosis of patients with NTG in the future.

## Figures and Tables

**Table 1:** PRISMA 2009 checklist.

Section/topic	#	Checklist item	Reported on page #
Title			
Title	1	Identify the report as a systematic review, meta-analysis, or both.	1

Abstract			
Structured summary	2	Provide a structured summary including, as applicable, background; objectives; data sources; study eligibility criteria, participants, and interventions; study appraisal and synthesis methods; results; limitations; conclusions and implications of key findings; systematic review registration number.	1

Introduction			
Rationale	3	Describe the rationale for the review in the context of what is already known.	1-2
Objectives	4	Provide an explicit statement of questions being addressed with reference to participants, interventions, comparisons, outcomes, and study design (PICOS).	2

Methods			
Protocol and registration	5	Indicate if a review protocol exists and if and where it can be accessed (e.g., Web address), and, if available, provide registration information including registration number.	NA
Eligibility criteria	6	Specify study characteristics (e.g., PICOS, length of follow-up) and report characteristics (e.g., years considered, language, and publication status) used as criteria for eligibility, giving rationale.	2-3
Information sources	7	Describe all information sources (e.g., databases with dates of coverage and contact with study authors to identify additional studies) in the search and date last searched.	2
Search	8	Present full electronic search strategy for at least one database, including any limits used, such that it could be repeated.	2
Study selection	9	State the process for selecting studies (i.e., screening and eligibility included in systematic review and, if applicable, included in the meta-analysis).	2-3
Data collection process	10	Describe method of data extraction from reports (e.g., piloted forms, independently, in duplicate) and any processes for obtaining and confirming data from investigators.	3
Data items	11	List and define all variables for which data were sought (e.g., PICOS and funding sources) and any assumptions and simplifications made.	3
Risk of bias in individual studies	12	Describe methods used for assessing risk of bias of individual studies (including specification of whether this was done at the study or outcome level) and how this information is to be used in any data synthesis.	3
Summary measures	13	State the principal summary measures (e.g., risk ratio and difference in means).	3
Synthesis of results	14	Describe the methods of handling data and combining results of studies, if done, including measures of consistency (e.g., *I* ^2^) for each meta-analysis.	3
Risk of bias across studies	15	Specify any assessment of risk of bias that may affect the cumulative evidence (e.g., publication bias, selective reporting within studies).	3
Additional analyses	16	Describe methods of additional analyses (e.g., sensitivity or subgroup analyses and metaregression), if done, indicating which were prespecified.	5

Results			
Study selection	17	Give numbers of studies screened, assessed for eligibility, and included in the review, with reasons for exclusions at each stage, ideally with a flow diagram.	5
Study characteristics	18	For each study, present characteristics for which data were extracted (e.g., study size, PICOS, and follow-up period) and provide the citations.	5
Risk of bias within studies	19	Present data on risk of bias of each study and, if available, any outcome level assessment (see Item 12).	6
Results of individual studies	20	For all outcomes considered (benefits or harms), present, for each study, (a) simple summary data for each intervention group and (b) effect estimates and confidence intervals, ideally with a forest plot.	6
Synthesis of results	21	Present results of each meta-analysis done, including confidence intervals and measures of consistency.	6, 10, 13
Risk of bias across studies	22	Present results of any assessment of risk of bias across studies (see Item 15).	5
Additional analysis	23	Give results of additional analyses, if done (e.g., sensitivity or subgroup analyses and metaregression [see Item 16]).	6, 10, 13

Discussion			
Summary of evidence	24	Summarize the main findings including the strength of evidence for each main outcome; consider their relevance to key groups (e.g., healthcare providers, users, and policy makers).	13-14, 16, 18
Limitations	25	Discuss limitations at study and outcome level (e.g., risk of bias) and at review level (e.g., incomplete retrieval of identified research and reporting bias).	18, 20
Conclusions	26	Provide a general interpretation of the results in the context of other evidence and implications for future research.	21

Funding			
Funding	27	Describe sources of funding for the systematic review and other support (e.g., supply of data); role of funders for the systematic review.	21

*From* [32].

For more information, visit http://www.prisma-statement.org/.

**Table 2:** Basic study data of the included studies.

Study	Country or region	Number of subjects (NTG/control)	Gender (male : female) (NTG/control)	Ethnic composition (NTG/control)	Mean age (years) (NTG/control)
[29]	Portugal	89/59	NA	NA	69.31 ± 12.1/71.44 ± 10.0
[43]	Portugal	62/48	NA	NA	70.4 ± 11/71.7 ± 9.5
[44]	Portugal	69/81	NA	NA	69.3 ± 11/64.6 ± 14
[45]	Portugal	63/55	NA	NA	69.7 ± 9.0/65.1 ± 11.0
[46]	Turkey	41/30	17 : 24/13 : 17	NA	56/53
[47]	UK	34/17	NA	NA	68.1 ± 8.7/65.2 ± 4.7
[48]	UK	25/26	NA	White 100%/White 100%	69.3 ± 8.9/65.7 ± 6.0
[49]	Italy	15/15	8 : 7/9 : 6	NA	64.7/65.8
[50]	Taiwan	13/25	9 : 4/24 : 1	NA	71.2/72.6
[51]	Italy	30/100^①^	16 : 14/46 : 54	NA	46.4/48.9
[52]	Japan	10/13	NA	NA	NA/NA
[53]	Germany	20/20	8 : 12/11 : 9	NA	61.3 ± 12.97/60.2 ± 18.78
[54]	Italy	44/40	24 : 20/22 : 18	Caucasian 100%/Caucasian 100%	64.45 ± 6.91/62.75 ± 7.37
[55]	USA	15/15	NA^②^	NA	61.4 ± 9.3/53.5 ± 8.8
[56]	Germany	15/15	6 : 9/6 : 9	NA	47 ± 8/41 ± 14
[57]	Switzerland	78/124	34 : 44/64 : 60	NA	NA
[58]	Germany	29/29	NA	NA	51 ± 10/44 ± 16
[30]	Germany	62/40	23 : 39/16 : 24	NA	57 ± 14/58 ± 9
[59]	Canada	23/28	NA	NA	64.8 ± 12.9/64.9 ± 12.4
[60]	Canada	42/35	15 : 27/16 : 19	NA	65.89 ± 11.15/61.87 ± 12.13
[61]	Belgium	28/22	NA	NA	72.8 ± 8.8/68.5 ± 8.9
[62]	Belgium	58/51	26 : 32/27 : 24	NA	70.9 ± 11.3/73.8 ± 13.3
[28]	China	60/44	31 : 29/20 : 24	NA	58.62 ± 13.05/57.41 ± 12.17

NA = not available.

^①^That study divided the normal controls into two groups according to whether they were older than 50 years. In this meta-analysis, two groups were combined to make normal control group age-matched to NTG group.

^②^That study only mentioned that there were 10 males and 20 females in all.

**Table 3 tab3:** Examining equipment and eye selection of the included studies.

Study	CDI equipment	Transducer frequency	Measurement body position
[29]	Antares CDI device (Siemens, Munich, Germany)	NA	NA
[43]	Antares CDI device (Siemens, Munich, Germany)	NA	NA
[44]	Antares CDI device (Siemens, Munich, Germany)	NA	NA
[45]	Antares CDI device (Siemens, Munich, Germany)	NA	NA
[46]	Toshiba SSA 240A scanner (Toshiba, Tokyo, Japan)	7.5 MHz	Supine
[47]	Acuson 128 machine (Mountain View, CA)	7.5 MHz	Supine
[48]	Acuson 128 machine (Mountain View, CA)	7.5 MHz	Supine
[49]	Asynchronous Hitachi Analyzer (Japan)	7.5 MHz	NA
[50]	Acuson 128 XP10 (Mountain View, CA)	7 MHz	Supine
[51]	QAD-l color Doppler unit (Quantum Medical Systems Inc., lssaquah, WA, USA)	7.5 MHz	NA
[52]	LOGIQ 500 system (GE Yokogawa Medical Systems, Tokyo, Japan)	7.5 MHz	Supine
[53]^①^	Sonoline Elegra Advanced System (Siemens, Erlangen, Germany)	6.5 MHz	Supine and sitting
[54]	DynaView II SSD-1700 (Aloka, Tokyo, Japan)	6 MHz	Supine
[55]	Siemens Quantum 2000 CDI system (Siemens Quantum Inc., Issaquah, WA, USA)	7.5 MHz	Sitting (lean against a chair ~60°)
[56]	Siemens Sonoline Sienna (Germany)	7.5 MHz	Supine
[57]	Siemens Quantum 2000 (Siemens Albis AG, Zurich, Switzerland)	7.5 MHz	Supine
[58]	Siemens Sonoline Sienna (Germany)	7.5 MHz	Supine
[30]	Siemens Sonoline Sienna (Germany)	7.5 MHz	Supine
[59]	Acuson 128 XP machine (Mountain View, California)	7 MHz	Supine
[60]^②^	System MedasonicII Transpect TCD (Medasonics, CA, USA)	2 MHz	Supine
[61]	Antares CDI device (Siemens, Munich, Germany)	NA	NA
[62]	Antares CDI device (Siemens, Munich, Germany)	7.5 MHz	Supine
[28]	HDI5000 (Philips Ultrasound, Bothell, WA, USA)	7.5 MHz	Supine

TCD = transcranial Doppler; NA = not available.

^①^This meta-analysis took the data in supine position of this study.

^②^Probe frequency in that study was used for transcranial Doppler.

**Table 4 tab4:** Quality-related data of the included studies.

Study	Inclusive criteria of NTG patients	Eye selection (NTG/control)	Type of treatment (topical)
[29]	Untreated IOP <21 mmHg Characteristic optic disc damage Glaucomatous visual field loss	Eye with greater glaucomatous damage/random	Beta blockers, 37 Prostaglandin analogs, 35 Carbonic anhydrase inhibitors, 31 Alpha-adrenergic agents, 7

[43]	Untreated IOP <21 mmHg Characteristic optic disc damage Glaucomatous visual field loss	Eye with greater glaucomatous damage/random	Beta blockers, 28 Prostaglandin analogs, 36 Carbonic anhydrase inhibitors, 20 Alpha-adrenergic agents, 6

[44]	Untreated IOP <21 mmHg Characteristic optic disc damage Glaucomatous visual field loss	Eye with greater glaucomatous damage/random	Beta blockers, 26 Prostaglandin analogs, 28 Carbonic anhydrase inhibitors, 24 Alpha agonists, 6

[45]	Untreated IOP <21 mmHg Characteristic optic disc damage Glaucomatous visual field loss	Eye with greater glaucomatous damage/random	Current topical IOP-lowering drugs (medicine not available)

[46]	Untreated IOP <21 mmHg Nerve fiber layer visual defects in each eye Bilateral symmetric glaucomatous-type optic disk cupping Glaucomatous visual field loss	Random/random	NA (35% with a first-time diagnosis of NTG)

[47]	Untreated IOP <22 mmHg Characteristic optic disc change Glaucomatous visual field loss	Eye with greater glaucomatous damage/random	Without any topical medication, 17 Using topical glaucoma medications with a wash-out period of 1 month, 17

[48]	Untreated IOP <22 mmHg Typical glaucomatous disc damage Glaucomatous visual field loss	Eye with worse field loss/random	Without any topical medication, 14 Using topical glaucoma medications with a wash-out period of 1 month, 11

[49]	Untreated IOP <18 mmHg Glaucomatous-type cupping of the optic disk Glaucomatous visual field loss	NA	NA

[50]	Untreated IOP <20 mmHg Glaucomatous optic nerve abnormality Glaucomatous visual field loss	NA	NA

[51]	Simple open angle glaucoma and normal pressure Glaucomatous appearance of optic disc Perimetric alterations (MD > 2dB, CPSD > 4dB)	NA	NA

[52]^①^	Untreated IOP <21 mmHg Progressive glaucomatous optic nerve damage (bilateral NTG)	Random/random	Using topical glaucoma medications with a wash-out period of 1 month

[53]	Untreated IOP <21 mmHg Abnormal thinning of the neuroretinal rim with glaucomatous cupping of the optic nerve head (cup-to-disc ratio 0.6) Glaucomatous visual field loss	Right eye preferential/right eye preferential	Using topical glaucoma medications with a wash-out period of 6 weeks

[54]	Untreated IOP <22 mmHg Functional defect of visual field (stage 1L according to Glaucoma Staging System 2)	Random eye in diseased eyes/random	Without any topical medication

[55]	Untreated IOP <22 mmHg Bilateral glaucomatous optic disc cupping Glaucomatous visual field loss	Right eye/right eye	NA

[56]	Untreated IOP <21 mmHg Glaucomatous excavation of the optic disc Glaucomatous visual field loss	Random/random	NA

[57]	Untreated IOP <21 mmHg A progressive optic neuropathy characterized by optic nerve head excavation Glaucomatous visual field loss	Random/random	Current topical IOP-lowering drugs (medicine not available)

[58]	Untreated IOP <21 mmHg Glaucomatous optic nerve head cupping Glaucomatous visual field loss	Random/random	Without any topical medication

[30]	Untreated IOP <21 mmHg Glaucomatous optic nerve head cupping Glaucomatous visual field loss	Random eye in diseased eyes/random	Without any topical medication or using topical glaucoma medications with a wash-out period of 3 weeks

[59]^②^	Untreated IOP <21 mmHg Glaucomatous optic nerve abnormality Glaucomatous visual field loss (bilateral NTG and NTG with one eye)	Right eye/right eye	Beta blockers, 8

[60]	Untreated IOP <21 mmHg Characteristic disc Glaucomatous visual field loss	Right eye/right eye	Current topical IOP-lowering drugs, 12 (medicine not available) Without any topical medication, 27 NA 3

[61]	Untreated IOP <21 mmHg Characteristic optic disc damage Glaucomatous visual field loss	Eye with greater glaucomatous damage/random	Current topical IOP-lowering drugs (medicine not available)

[62]	Untreated IOP <21 mmHg Characteristic optic disc change Glaucomatous visual field loss	Eye with greater glaucomatous damage/random	Current topical IOP-lowering drugs (medicine not available)

[28]	Untreated IOP <21 mmHg Glaucomatous optic nerve appearance Glaucomatous visual field loss	Random eye in diseased eyes/random	Without any topical medication

NA = not available.

^①^In that study, all NTG patients selected were with bilateral NTG. That study aimed to investigate the effects of nipradilol on retrobulbar hemodynamic. NTG patients and normal controls were given nipradilol in one random eye and placebo in the other. This meta-analysis only used the data of the eye which would be instilled with nipradilol before the NTG patients and normal controls were given nipradilol.

^②^That study provided outcome measurements data of both eyes, but this meta-analysis only chose the data of right eye according to the eye selection method of several of the other included studies. The NTG patients in that study were composed of 22 patients with bilateral normal tension glaucoma and 2 patients with normal tension glaucoma who had only one eye (one had only right eye and one had only left eye).

**Table 5:** Outcome measures data of the included studies.

Study	Results of PSV (cm/s) (NTG/control)	Results of EDV (cm/s) (NTG/control)	Results of RI (NTG/control)
[29]	OA: 33.6 ± 11.2/40.1 ± 16.9	OA: 6.79 ± 3.32/7.35 ± 4.36	OA: 0.80 ± 0.07/0.82 ± 0.07
	CRA: 10.32 ± 3.58/12.0 ± 4.38	CRA: 2.87 ± 1.08/3.10 ± 1.12	CRA: 0.71 ± 0.08/0.73 ± 0.07
	NPCA: 8.58 ± 2.69/11.08 ± 3.76	NPCA: 2.92 ± 0.99/3.53 ± 1.53	NPCA: 0.65 ± 0.07/0.68 ± 0.08
	TPCA: 9.12 ± 2.81/11.0 ± 3.87	TPCA: 3.06 ± 1.09/3.45 ± 1.36	TPCA: 0.66 ± 0.08/0.68 ± 0.08

[43]	OA: 35.3 ± 10.7/41.7 ± 18.8	OA: 7.5 ± 3.5/8.3 ± 5.3	OA: 0.79 ± 0.07/0.81 ± 0.07
	CRA: 10.6 ± 4.0/12.1 ± 4.0	CRA: 2.8 ± 0.98/3.2 ± 1.2	CRA: 0.71 ± 0.08/0.73 ± 0.07
	NPCA: 8.2 ± 2.6/11.6 ± 4	NPCA: 2.8 ± 0.9/3.6 ± 1.6	NPCA: 0.65 ± 0.07/0.68 ± 0.08
	TPCA: 9.2 ± 3.1/11.5 ± 3.9	TPCA: 3.1 ± 1.2/3.6 ± 1.5	TPCA: 0.66 ± 0.07/0.68 ± 0.08

[44]	OA: 31.6 ± 10/37.7 ± 17	OA: 5.81 ± 3.7/6.85 ± 3.9	OA: 0.82 ± 0.1/0.82 ± 0.1
	CRA: 10.7 ± 3.4/11.9 ± 4.3	CRA: 2.96 ± 1.1/3.20 ± 1.2	CRA: 0.71 ± 0.1/0.72 ± 0.1
	NPCA: 9.51 ± 2.8/9.29 ± 2.7	NPCA: 3.2 ± 1.0/3.13 ± 1.1	NPCA: 0.66 ± 0.1/0.66 ± 0.1
	TPCA: 9.20 ± 2.7/8.97 ± 2.4	TPCA: 3.06 ± 0.9/3.08 ± 0.9	TPCA: 0.66 ± 0.1/0.65 ± 0.1

[45]	OA: 32.9 ± 10/39.7 ± 17	OA: 6.03 ± 3.8/7.96 ± 4.6	OA: 0.82 ± 0.1/0.80 ± 0.1
	CRA: 10.6 ± 3.5/12.9 ± 4.4	CRA: 2.88 ± 0.9/3.55 ± 1.3	CRA: 0.71 ± 0.1/0.72 ± 0.1
	NPCA: 9.52 ± 3.0/10.8 ± 4.1	NPCA: 3.33 ± 1.2/3.81 ± 1.7	NPCA: 0.64 ± 0.1/0.65 ± 0.1
	TPCA: 9.54 ± 3.3/11.1 ± 3.7	TPCA: 3.22 ± 1.2/3.80 ± 1.4	TPCA: 0.66 ± 0.1/0.65 ± 0.1

[46]	OA: NA	OA: NA	OA: 0.72 ± 0.07/0.69 ± 0.06
	CRA: NA	CRA: NA	CRA: 0.60 ± 0.06/0.53 ± 0.07
	NPCA: NA	NPCA: NA	NPCA: 0.61 ± 0.07/0.53 ± 0.06
	TPCA: NA	TPCA: NA	TPCA: 0.61 ± 0.08/0.54 ± 0.06

[47]	OA: 30.4 ± 7.6/30.1 ± 9.9	OA: 6.6 ± 2.9/8.4 ± 2.9	OA: 0.77 ± 0.08/0.72 ± 0.05
	CRA: 12.8 ± 9.1/13.4 ± 6.3	CRA: 1.9 ± 1.9/2.6 ± 1.6	CRA: 0.87 ± 0.09/0.80 ± 0.08
	NPCA: NA	NPCA: NA	NPCA: NA
	TPCA: NA	TPCA: NA	TPCA: NA

[48]	OA: 31.5 ± 8.1/30.8 ± 10.6	OA: 7.1 ± 2.9/8.3 ± 3.1	OA: 0.77 ± 0.08/0.73 ± 0.05
	CRA: 12.4 ± 7.9/13.0 ± 6.2	CRA: 1.7 ± 1.8/3.0 ± 1.6	CRA: 0.86 ± 0.09/0.77 ± 0.09
	NPCA: NA	NPCA: NA	NPCA: NA
	TPCA: NA	TPCA: NA	TPCA: NA

[49]	OA: 32.961 ± 11.793/36.646 ± 25.604	OA: NA	OA: 0.717 ± 0.074/0.789 ± 0.070
	CRA: NA	CRA: NA	CRA: NA
	NPCA: NA	NPCA: NA	NPCA: NA
	TPCA: NA	TPCA: NA	TPCA: NA

[50]	OA: 28.9 ± 10/30.9 ± 8.4	OA: 4.3 ± 1.5/5 ± 2.5	OA: 0.84 ± 0.07/0.84 ± 0.05
	CRA: 9.3 ± 2.8/9.6 ± 3.5	CRA: 2.09 ± 1.2/2.07 ± 0.78	CRA: 0.78 ± 0.1/0.77 ± 0.07
	NPCA: 7.4 ± 3.6/7.7 ± 2.9	NPCA: 2.1 ± 1.7/1.8 ± 0.9	NPCA: 0.72 ± 0.11/0.77 ± 0.07
	TPCA: 7.5 ± 2.4/8.4 ± 2.6	TPCA: 2.1 ± 0.74/2.1 ± 0.98	TPCA: 0.71 ± 0.07/0.75 ± 0.08

[51]	OA: 37.320 ± 1.729	OA: NA	OA: 0.708 ± 0.008
	CRA: NA	CRA: NA	CRA: NA
	NPCA: NA	NPCA: NA	NPCA: NA
	TPCA: NA	TPCA: NA	TPCA: NA

[52]	OA: 28.4 ± 10.8/35.8 ± 8.4	OA: 7.71 ± 3.73/8.03 ± 2.40	OA: 0.725 ± 0.084/0.776 ± 0.035
	CRA: 9.89 ± 3.31/11.3 ± 3.1	CRA: 2.88 ± 1.17/4.18 ± 1.06	CRA: 0.711 ± 0.046/0.627 ± 0.038
	NPCA: 9.03 ± 3.23/8.13 ± 1.85	NPCA: 3.18 ± 1.40/3.43 ± 1.10	NPCA: 0.651 ± 0.061/0.584 ± 0.059
	TPCA: 8.76 ± 2.53/10.2 ± 2.8	TPCA: 2.95 ± 0.93/3.95 ± 1.27	TPCA: 0.662 ± 0.055/0.614 ± 0.060

[53]	OA: 35.50 ± 9.84/31.40 ± 13.42	OA: 7.80 ± 3.58/7.60 ± 5.81	OA: 0.77 ± 0.09/0.78 ± 0.09
	CRA: 9.80 ± 3.13/11.10 ± 2.24	CRA: 2.7 ± 1.34/3.30 ± 1.79	CRA: 0.73 ± 0.09/0.70 ± 0.09
	NPCA: NA	NPCA: NA	NPCA: NA
	TPCA: NA	TPCA: NA	TPCA: NA

[54]	OA: 25.43 ± 5.34/32.90 ± 6.17	OA: 5.87 ± 1.17/11.41 ± 2.30	OA: 0.76 ± 0.03/0.64 ± 0.03
	CRA: 11.41 ± 1.96/12.14 ± 1.80	CRA: 3.72 ± 0.77/5.67 ± 1.11	CRA: 0.66 ± 0.07/0.52 ± 0.08
	NPCA: NA	NPCA: NA	NPCA: NA
	TPCA: NA	TPCA: NA	TPCA: NA

[55]	OA: 34.9 ± 9.3/35.9 ± 9.1	OA: 4.8 ± 2.3/9.9 ± 5.2	OA: 0.87 ± 0.06/0.73 ± 0.08
	CRA: 8.5 ± 1.8/8.7 ± 2.2	CRA: 1.2 ± 1.1/2.5 ± 1.1	CRA: 0.86 ± 0.12/0.72 ± 0.08
	NPCA: NA	NPCA: NA	NPCA: NA
	TPCA: NA	TPCA: NA	TPCA: NA

[56]	OA: 30.16 ± 10.21	OA: 7.97 ± 2.84	OA: 0.78 ± 0.07
	CRA: 30.75 ± 7.64	CRA: 6.85 ± 3.35	CRA: 0.73 ± 0.05
	NPCA: NA	NPCA: NA	NPCA: NA
	TPCA: NA	TPCA: NA	TPCA: NA

[57]	OA: 35.87 ± 9.71/38.90 ± 5.57	OA: 7.68 ± 2.65/9.15 ± 2.23	OA: 0.78 ± 0.07/0.75 ± 0.07
	CRA: 9.24 ± 1.77/11.10 ± 1.11	CRA: 2.29 ± 0.88/3.23 ± 0.78	CRA: 0.75 ± 0.07/0.70 ± 0.06
	NPCA: 10.46 ± 2.65/11.27 ± 1.11	NPCA: 2.81 ± 0.88/3.57 ± 0.89	NPCA: 0.71 ± 0.08/0.68 ± 0.06
	TPCA: 9.77 ± 1.77/11.12 ± 1.11	TPCA: 2.91 ± 0.88/3.57 ± 0.89	TPCA: 0.70 ± 0.08/0.68 ± 0.06

[58]	OA: 29.2 ± 9.4/36.0 ± 8.3	OA: 8.1 ± 3.3/8.1 ± 3.7	OA: 0.73 ± 0.06/0.78 ± 0.07
	CRA: 7.2 ± 1.6/9.8 ± 2.2	CRA: 1.8 ± 0.9/3.2 ± 0.7	CRA: 0.75 ± 0.10/0.67 ± 0.07
	NPCA: 6.8 ± 1.2/8.5 ± 1.7	NPCA: 2.1 ± 0.8/3.5 ± 0.6	NPCA: 0.70 ± 0.10/0.59 ± 0.05
	TPCA: 7.3 ± 1.3/8.4 ± 1.9	TPCA: 2.2 ± 1.0/3.5 ± 0.8	TPCA: 0.70 ± 0.12/0.58 ± 0.06

[30]	OA: 30.8 ± 8.8/32.8 ± 9.1	OA: 7.8 ± 3.2/7.4 ± 3.5	OA: 0.75 ± 0.07/0.78 ± 0.07
	CRA: 7.6 ± 1.8/9.3 ± 1.9	CRA: 2.0 ± 0.9/2.9 ± 0.9	CRA: 0.74 ± 0.09/0.69 ± 0.06
	NPCA: 6.9 ± 1.5/7.6 ± 1.8	NPCA: 2.2 ± 0.9/2.7 ± 0.7	NPCA: 0.69 ± 0.09/0.63 ± 0.07
	TPCA: 7.3 ± 2.3/7.5 ± 1.7	TPCA: 2.3 ± 1.0/2.8 ± 0.7	TPCA: 0.69 ± 0.1/0.62 ± 0.07

[59]	OA: NA	OA: NA	OA: NA
	CRA: 12.0 ± 2.9/14.5 ± 4.7	CRA: 3.2 ± 1.3/4.5 ± 1.5	CRA: 0.74 ± 0.08/0.69 ± 0.08
	NPCA: 9.0 ± 1.9/9.6 ± 1.9	NPCA: 2.8 ± 1.0/3.4 ± 1.0	NPCA: 0.70 ± 0.09/0.66 ± 0.09
	TPCA: 9.0 ± 2.2/10.0 ± 2.1	TPCA: 2.9 ± 0.85/3.8 ± 1.3	TPCA: 0.68 ± 0.09/0.62 ± 0.09

[60]	OA: 35.35 ± 7.78/43.86 ± 7.81	OA: 8.96 ± 2.66/11.92 ± 2.60	OA: NA
	CRA: NA	CRA: NA	CRA: NA
	NPCA: NA	NPCA: NA	NPCA: NA
	TPCA: NA	TPCA: NA	TPCA: NA

[61]	OA: 36.8 ± 14.1/45.8 ± 16.0	OA: 7.4 ± 3.1/10.4 ± 6.1	OA: 0.79 ± 0.08/0.78 ± 0.08
	CRA: 9.1 ± 3.4/14.0 ± 3.5	CRA: 2.9 ± 0.9/3.7 ± 1.2	CRA: 0.67 ± 0.08/0.73 ± 0.08
	NPCA: 10.1 ± 3.5/12.8 ± 4.5	NPCA: 3.4 ± 1.3/4.3 ± 2.0	NPCA: 0.66 ± 0.07/0.67 ± 0.07
	TPCA: 10.1 ± 4.0/12.8 ± 3.9	TPCA: 3.4 ± 1.5/4.3 ± 1.7	TPCA: 0.68 ± 0.07/0.66 ± 0.08

[62]	OA: 30.25 ± 10.5/36.63 ± 14.5	OA: 5.24 ± 2.3/6.55 ± 4.1	OA: 0.82 ± 0.1/0.82 ± 0.1
	CRA: 10.31 ± 3.1/11.16 ± 4.6	CRA: 2.92 ± 1.0/3.00 ± 1.3	CRA: 0.71 ± 0.1/0.73 ± 0.1
	NPCA: 9.22 ± 2.7/10.29 ± 3.3	NPCA: 3.16 ± 1.0/3.28 ± 1.3	NPCA: 0.65 ± 0.1/0.67 ± 0.1
	TPCA: 9.07 ± 2.3/10.1 ± 3.5	TPCA: 3.00 ± 0.8/3.31 ± 1.4	TPCA: 0.66 ± 0.1/0.66 ± 0.1

[28]	OA: 31.50 ± 8.40/39.55 ± 7.24	OA: 8.37 ± 2.93/12.64 ± 4.36	OA: 0.73 ± 0.06/0.69 ± 0.07
	CRA: 10.04 ± 3.09/12.36 ± 2.84	CRA: 3.49 ± 0.99/4.77 ± 1.77	CRA: 0.66 ± 0.07/0.61 ± 0.07
	NPCA: NA	NPCA: NA	NPCA: NA
	TPCA: NA	TPCA: NA	TPCA: NA

NTG = normal tension glaucoma; IOP = intraocular pressure; PSV = peak systolic velocity; EDV = end diastolic velocity; RI = resistive index; OA = ophthalmic artery; CRA = central retinal artery; NPCA = nasal short posterior ciliary artery; TPCA = temporal short posterior ciliary artery; NA = not available.
